# Novel molecular insights and new therapeutic strategies in osteosarcoma

**DOI:** 10.1186/s12935-018-0654-4

**Published:** 2018-10-16

**Authors:** Babak Otoukesh, Bahram Boddouhi, Mehdi Moghtadaei, Peyman Kaghazian, Maria Kaghazian

**Affiliations:** 1 Bone and Joint Reconstruction Research Center, Shafa Orthopedic Hospital, Iran University of Medical Sciences, Tehran, 1445613131 Iran; 2 Department of Orthopedic and Traumatology, Universitätsklinikum Bonn, Bonn, Germany; 3 Department of Biology, Jundishapur University of Medical Sciences, Ahvaz, Iran

**Keywords:** OS, Molecular targets, Stem cells, Molecular mechanism, Cancer therapy

## Abstract

Osteosarcoma (OS) is one of the most prevalent malignant cancers with lower survival and poor overall prognosis mainly in children and adolescents. Identifying the molecular mechanisms and OS stem cells (OSCs) as new concepts involved in disease pathogenesis and progression may potentially lead to new therapeutic targets. Therefore, therapeutic targeting of OSCs can be one of the most important and effective strategies for the treatment of OS. This review describes the new molecular targets of OS as well as novel therapeutic approaches in the design of future investigations and treatment.

## Background

Osteosarcoma (OS) is one of the most prevalent malignant cancers in the bone, which is seen mainly in children and adolescents. However, a second incidence peak can be occurred in the elderly [1–6]. OS often originates from long bones including the distal femur (30%) and proximal tibia (15%), as well as proximal humerus (15%), [6]. OS comprises almost 20% of all cases of benign and malignant bone neoplasia [4–6]. Moreover, about 20% of patients with OS could develop metastatic OS and the overall prognosis for these subjects was revealed to be poor with a 10–50% overall survival rate. Despite aggressive chemotherapy surgery in patients with localized OS, 30–40% of which experience relapse. Based on the data provided in several large series, the 5-year survival rate has been estimated to be between 23 and 29% [7].

The majority of the patients show relapses due to metastases to the lungs as the primary site with poor 5-year survival rates [3, 8]. Additionally, the 5-year survival rates are accordingly estimated to be 50–60% for patients suffering from relapse [9]. Nevertheless, the survival rates for patients with metastatic and recurrent disease is less than 30%, and has not substantially changed; therefore, a deep understanding of functional mechanisms is needed for the development of new anti-cancer strategies, where many molecular agents are involved, not only in tumor growth but also in the conditioning metastasis [10, 11]. The combination of surgery with chemotherapy has been considered as primary therapeutic strategies for OS. Nevertheless, resistance to chemotherapy is commonly seen, leading to a recurrence of the tumor [4]. Although most researchers believe that OS have originated from mesenchymal stem cells (MSCs), osteoprogenitor cells, but its origin is still unclear [4, 12, 13]. The importance of OS stem cells (OSCs) has been highlighted recently, which are linked to resistance, recurrence, and metastasis through self-renewal and differentiation. Furthermore, it has been revalued that cancer stem cells (CSCs) are more malignant as compared with differentiated cancer cells [4, 14]. However, further research is needed to identify the biology of the OSCs during tumor progression to develop robust diagnostic and effective therapies by targeting these cells.

Our accurate understanding of the in vivo biology of stem cells is very important because its precise understanding can be very promising for expanding new in vitro experiments, correcting the limitations and practical errors.

## Identifying OS stem cell populations as therapeutic targets

The discovery of oncogenes and tumor suppressor genes has led to a better understanding of the genetic nature of the cancer; therefore, it can be claimed that the theory of cancer stem cells is a new concept that has been the mainstay of cancer studies in the past decade. This hypothesis has attracted significant support for OSl studies; hence, several studies have evaluated a number of human and mouse OS cells that were capable of expressing stem cell marker responsible for tumor-forming ability [14].

**Fig. 1:**
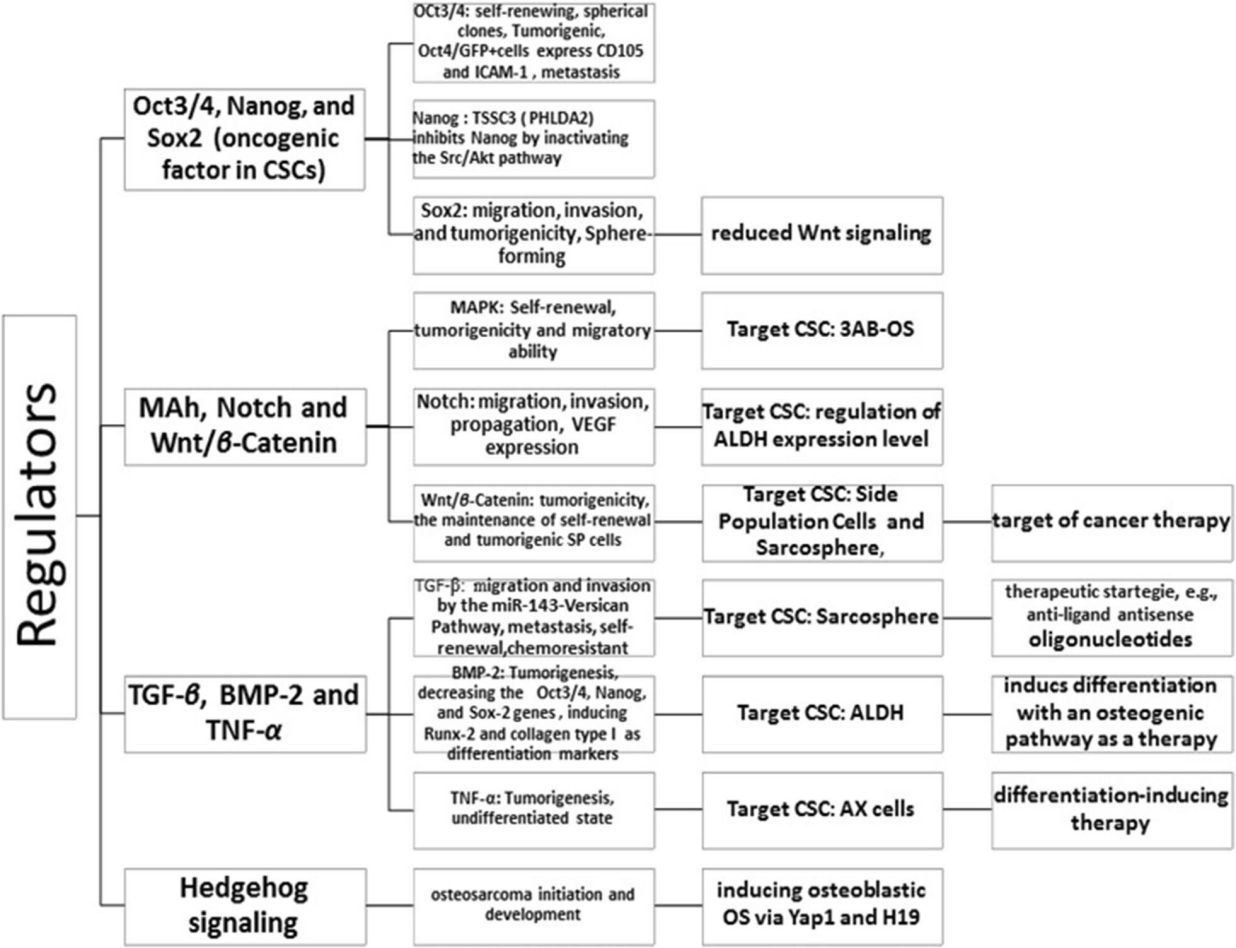
Master regulators of bone sarcoma stem cells in a glance as reported by previous studies

It has been revealed that human CSCs play a key role in tumor initiation, relapse, drug resistance, invasion, and metastasis. Thus, therapeutic targeting of OSCs can be one of the most important opportunities for the development of cancer research. With this regard, these strategies require identifying, understanding, and isolating OSCs [15]. Many markers have been identified for the isolation of OSCs, but some of them are highly effective including CD133, CD117, Stro-1 and ALDH [4, 16]. However, these markers have had partial success in isolating cancer stem cells (CSCs) from various cancers. Markers of CSCs in OS are categorized in Fig. 1 based on the available data. Markers for isolation of OSCs, their functional role and clinical relevance are summarized in Table 1. The regulation of intracellular markers (i.e., aldehyde dehydrogenase [ALDH]), cell surface markers (i.e., CD133, CD117, CD44, CD 271, Sca-1), stemness genes (i.e., Nestin, Sox2, OCT3/4, and Nanog), phenotypic evaluation (e.g., sphere forming assay), side population (SP) cells are mostly provided for evaluating and isolating OSCs in tumors [17–19].

**Table 1:** Markers of bone sarcoma stem cells

Marker	Function, clinical relevance	References
Sarcosphere	Chemoresistance (e.i., doxorubicin and cisplatin etc.,) overexpressing Oct3/4, and Stat3	[42, 96]
CD133	Sphere formation, self-renewal ability, multipotency, tumorigenicity, inclusion of SP cells, upregulating stemness genes of Nestin, Sox2, OCT3/4, and Nanog	[15, 20, 21, 25, 26, 37, 48, 75, 106]
CD117/Stro-1	Chemoresistance features, higher level of self-renewal, tumorigenicity, multipotent invasive, metastasis, inducing ABCG2 and CXCR4 overexpression	[24, 39, 71]
ALDH	Differentiation, self-renewal ability, tumorigenicity, metastatic potential, over-expressing Oct3/4, Nanog, Sox2, and Stat3	[32, 33, 84]
CD271	Tumorigenicity, self-renewal, differentiation, and the advantage of sarcospheres formation	[27, 28]
Side population	Clonogenicity, drug resistance, self-renewal capacity, and tumor-initiating capacity in CSCs and invasiveness	[44, 45, 83]
CD44	*Sphere*-*forming*, invasiveness, Tumorigenic and metastatic property	[39]
MSC antigen Sca-1	Overexpression of Sca-1 and Sox2 cells can be capable of self-renewal in OS-stem cells	[96, 104]
CD248	Tumorigenicity and invasiveness	[26]
ABCA5	Considered as putative biomarkers and overexpressed in spheres	[43]
CD47	Its CD47 blockade was linked to inhibition of tumor growth., invasion, prognostic factor, and immunotherapeutic target	[34, 35]
Oct3/4	Tumorigenic, self-renewal capability and *metastatic potential*	[33, 84]
ABCG2 and CXCR4	Metastasis-associated marker CXCR4 and drug-resistance marker ABCG2	[38, 39, 71]
Sox2	Tumorigenicity, Sphere formation, invasiveness, and *cancer* cell *migration*	[26, 104]
Nestin	CD133, Oct3/4, Sox2, Nanog, nestin Coexpression of Nes^+^/CD133^+^ cells; presence of cells with a stem-like phenotype	[26, 48]

### Markers for isolation of OSCs

#### CD133

The CD133 molecule is a well-known stem cell marker of normal and cancerous tissues. CD133 offers an exciting accessibility to, not only separate stem cells from tissues (i.e., bone marrow), [20–22], but also to isolate cancer stem cells from tumors by using monoclonal antibodies against CD133 [22, 23]. It has been shown that CD133 expression can be induced by chemotherapy, where its expression is directly related to the increased expression of miR-133a, indicating the induction of CSC through chemotherapy. Therefore, with regard to the above, it seems that CD133 should be considered as a potential therapeutic target in OS [24].

In a study by Adhikari et al. it was suggested that CD117 and Stro-1 expressed in spheres and doxorubicin-resistant OS cells. It has found that CD117-Stro-1 double-positive OSCs are detectable in mouse and human OS cell lines and primary cultures, where their presence was associated with high invasive, metastatic, and drug resistance features, as well as higher level of self-renewal. Moreover, they have been revealed to be enriched in CXCR4 (20–90%) and ABCG2 (60–90%), which are recognized as metastasis-associated markers and drug-resistance markers [4, 25]. Increased expression levels of Oct-4, NANOG, and the CXCR4 have been demonstrated in CD133^+^ cells, where CD133^+^ cells were found to be highly active in invasion and migration while comparing with CD133^−^ cells. It has been revealed that overexpression of CD133 in OS tissues was linked to an elevated risk of lung metastasis and shorter survival time in patients suffering from OS [26]. Evidence for the presence of CSC has been found in early human bone sarcomas, suggesting CD133 as a marker for their detection. CSCs CD133 derived from human sarcoma could be targeted for therapeutic strategies, and may be appropriately addressed in the prognosis of the disease. It is noteworthy, CD133(+) subpopulation formed sarcospheres, where sarcospheres were found to be positive for stemness genes expression of Nestin, Sox2, OCT3/4, and Nanog;on the other hand, sarcospheres revealed self-renewal, and differentiation abilities, as well as high tumorigenicity in vivo [27].

#### CD271

CD271 has been defined to play a substantial role in OSCs as an effective marker, where CD271^+^ cells exhibited many stem-like properties, such as tumorigenicity, self-renewal, differentiation, and the advantage of sarcospheres formation, as well as drug resistance [28, 29].

#### ALDH

High ALDH has been previously applied as a marker for identifying tumorigenic cell fractions in many kinds of malignancies and reveled to be associated with tumorigenic cell fractions (higher levels of tumorigenicity), differentiation and self-renewal as well as metastatic potential in OS cell lines [4, 30]. ALDH activity is upregulated in cancer stem cells, where is shown as a marker for cancer stem cells [31–33]. The inhibition of ALDH activity by applying disulfiram has led to a reduction in cell proliferation, consequently, it suggests direct targeting of CSC genotypes [24, 34].

### Other OSC phenotype-associated factors

#### CD47 as immunotherapeutic target

Previously, it has been found that CD47 could regulate osteoclastogenesis by regulation of NO production, while its disruption was associated with a reduced level of metastasis in bone tumor [35].

CD47 protein expression has been revealed to be markedly expressed in OS tissues when comparing with control osteoblastic cells as normal cells and adjacent tissues. It has been reported that CD47 blockade was linked to tumor growth inhibition in the xenograft models of OS, resulting macrophage phagocytosis of tumorous cells with potential characteristic for therapeutic strategies of OS (immunotherapeutic), [36].

CBX3 and ABCA5 as putative biomarkers of TSCs and/or OS, ABCG2 as a novel target.

ATP-binding cassette transporters (ABC transporters) are classified into a family of transporter proteins, which are involved in multidrug resistance (MDR) [37]. Overexpression of ABCG2 transporter has been initially found by FACS analyses in MG63, SAOS2 and U2OS as human sarcoma cell lines [38]. ABCG2 expression was previously applied to detect drug resistant side population (SP) or tissue-specific stem cells (TSCs) in many kinds of malignancies, such as OS [39]. It should be taken into consideration that conserved expression of ABCG2 can mostly mediate the SP phenotype in stem cells, and introduced as a promising biomarker of CSCs. ABCG2+ tumor cells have been potentially presented an unparalleled population of CSCs [39].

The expression of IGF1R has been recently appeared to be linked to ABCG2 and CD44 expression levels in OS, suggesting their conventional prognostic utility and potential as therapeutic targets with IGF1R for OS [40]. Over the past 10 years, a novel ABC transporter inhibitors has been provided to counteract the high toxicity of effective doses used to inhibit ABC transporter activity [36, 40], which promising preclinical findings have also been shown in HGOS cells [41–43]. CBX3 and ABCA5 as putative biomarkers of TSCs and/or OS, ABCG2 as a novel target.

Saini et al. found that CD326 CD24, and CD44 have overexpressed in TSC-enriched as compared with un-enriched cultures, whereas overexpression of ABCG2 and CBX3 were also found. They suggested two putative biomarkers (CBX3 and ABCA5) for OSCs. Furthermore, in aforementioned study, RHPS4, vincristine, and 5-Aza-C have been potentially suggested to be therapeutic agents against TSC-enriched OS cultures; however, it should be taken into consideration that they need to be tested in vivo for their therapeutic approach [44].

#### Sca-1 stem cell antigen as effective OSC marker

A study by Basu-Roy et al. found that depletion of Sox2 in OS cells demonstrated a reduced level of osteosphere formation and Sca-1 expression, coupled with an escalation of differentiation into mature bone osteoblasts formation by activating Wnt signaling [14].

#### Side population (SP) cells

SP has been revealed to be associated with clonogenicity, drug resistance, self-renewal, and tumor-initiating capacity in CSCs. SP cells exhibited drug resistance, invasiveness and clonogenicity while comparing with non-SP cells. Overall, SP cells have been shown to be involved in metastasis and recurrence in Ewing’s sarcoma SK-ES-1 cells as a potential target for clinical therapy [45, 46]. OS SP cells has been demonstrated stem-like features. SP cell-derived sarcospheres have reveled overexpression of CD133 and Oct-3/4A, where these cells found to be associated with resistance to different drug therapies. SP cells with overexpression of CD248 cells have been shown to be implicated tumorigenicity and invasiveness, which CD248 was suggested as a therapeutic target. Endosialin overexpression in OS SP cells has been raised to be a marker for SP cells purification and construction of anti-cancer drugs [47].

#### Nestin identification in CSCs from OS

Zambo et al. found a relationship of the nestin expression level in high-grade OSs with the clinical outcomes for OS [48]. The coexpression of Nes^+^/CD133^+^ cells in OS cell lines has been proven based on the use of immunodetection studies, indicating the probable presence of cells with a stem-like phenotype [49]. On the contrary, a study reported that nestin has been overexpressed at the transcript level in CHA59 spheres as competed to monolayers monolayer cells. This pattern has been also found for CHA59 cells at the protein level; whereas, similar patterns were not revealed based on the use of Saso-2 and HuO9 cells. It is worth noting that both spheres and adherent cells have been identified to be nestin-negative for the Saos-2 cell line [44], while nestin overexpression has been reported in the OS cell lines by Veselska et al. based upon the use of immunofluorescence [49]. Heterogeneous expression of nestin has been previously highlighted suggestive of the tumor heterogeneity [42, 49].

Another study revealed that nestin mRNA expression was detected in sphere-forming subpopulations, whereas adherent subpopulations belonging to the same cell lines were found as nestin negative. CD133(+) subpopulation formed sarcospheres, which played a role in initiating and sustaining tumor growth as well as stemness genes expression of Nestin, Sox2, OCT3/4, and Nanog [27].

### MicroRNA therapeutic targets

MicroRNAs (miRNA) can participated in modulating of multiple genes and the CSCs functions. This can be a very important strategy for targeting CSC. So that, they are searched for cancer treatment purposes. The miRNA are controversially contributing to the survival or prevention of the CSC; nevertheless, it should be taken into consideration that inhibiting or stimulating the expression of specific miRNAs in a variety of cancers can be considered as therapeutic strategies [50–52]. New anticancer therapies are performed to either reduce or increase their expression level by manipulating tumor suppressor or tumorigenic miRNAs [51].

MiR-133a has been found to regulate the cell invasion in the SaOS2 CD133 high population and cell invasion was greatly reduced when miR-133a was silenced with locked nucleic acid (LNA); while upregulation of CD133 and miR-133a have been markedly correlated with poor prognosis. The miR-133a has been suggested with concurrent chemotherapy as a new strategy that can be used to target several regulatory pathways involved in metastasis in OS [53].

Downregulation of miR26a has been demonstrated in OS CSCs by Lu et al. [54]. They indicated that higher expression of miR26a was linked to lung metastasis. Moreover, overexpression of miR-26a has participated to decrease CSC marker expression and inhibited sphere-forming, and ALDH function, as well as OS tumor cells via the repression of Jagged1. Another study by Golbakhsh et al. suggested that expression of MiR-182 and MiR-183 may be linked to progression and metastasis in patients suffering from OS [51]. Therefore, the current data supports an regulatory role for miR-26a/Jagged1/Notch pathway in OS CSCs, which regulated stemlike traits. Thereby, miR-26a, Jagged1, and Notch pathway being explored as therapeutic target for OS.

Current results have also attributed a role to miR-29b-1 in inducing CSCs self-renewal, proliferation, chemosensitivity as well as inhibiting stemness properties of CSCs, where a set of markers are involved in this event including CD133, Nanog, N-Myc, Oct3/4, and Sox2 as stem cell markers; and anti-apoptotic Bcl-2 and IAP-2 markers; E2F1, E2F2 and CCND2 markers as cell cycle-associated molecules [51]. Therefore, miR-29b-1 it has been suggested as a new therapeutic strategy for OS. IncRNA HIF2PUT was found to be overexpressed on OS stem cells in vitro, where its overexpression has been associated with some properties in MG63 OS cells including, inhibition of cell migration, proliferation, and sphere-forming capacity as well as a reduction in the number of CD133 positive cells. IncRNA HIF2PUT has been considered to be a new regulatory agent, which may play its functional role for regulating hypoxia-inducible factor-2α (HIF-2α), suggesting its potential as a therapeutic target of OS [55].

### Sphere-forming assays

Today, sphere-forming assay has increasingly been applied to study the stemness and enrichment of CSCs or tumor initiating cells (TICs) [56]. This assay has been involved in generating and maintaining CSCs/TICs with high tumorigenesis in many kinds of malignancies including, rectum, colon, breast cancer, bone, etc. [57].

It has been indicated that sphere-forming stem-like cells were potentially linked to CDDP and DXR resistance. Based on the data presented in sphere cells, DNA repair enzyme genes, including MLH1 and MSH2, were reported to be markedly overexpressed in MG63 and HTB166 cell lines, indicating the potential relationship of drug resistance of human sarcoma cell lines with elevated DNA repair enzyme. As a matter of fact, it has been revealed that sphere-forming stem-like cells can be associated with the higher efficacy of chemotherapy agents in sarcomas [58].

A previous study indicated that the isolated CSCs from spheres had demonstrated mesenchymal stem cell characteristics such as overexpressed markers of Nanog, Oct 3/4 and the ABC transporters P-glycoprotein. They also revealed that MNNG/HOS OS cell line with stem-like cell was associated with tumorigenicity and elevated resistance against cancer treatment. This resistant has been found to be linked to upregulation of the BCRP and ABC transporters P-glycoprotein [59]. It is worth noting that clarifying the role of CSCs derived from patients’ in therapy response is substantial for establishing new therapeutic strategies. A study confirmed stem-like cell population in four OS cell lines (Hu09 cells, Saos-2 cells, OS99-1 cells, and MG63 cells) based on the use of sphere forming assay and expression level of Oct3/4 A and Nanog as markers for stem cell, indicating evidence for origin of cancer derived from stem cells. In addition, they found that Oct3/4B was markedly expressed in Hu09 cell line when comparing with MG63 and OS99-1 cells, with high metastasis, indicating Oct3/4B tumor metastatic potential, where sarcogenesis has been also revealed [57].

Another study by Palmini et al. provided a primary finite cell line of the small cell OS (SCO) from which the CSCs was obtained by applying the sphere formation evaluation, indicating the presence of CSCs in human primary SCO. They provided cell line of CSCs as new in vitro model in evaluating SCO Biology [60].

TGFβ1 or hypoxic environment have been found to be remarkably associated with spheres forming in MNNG/HOS and MG63 as OS cell lines, where TGF-β1 signaling and a hypoxic condition were triggered self-renewal properties in non-stem cell and can be involved in neovasculogenesis, tumorigenicity, and metastasis, as well as chemoresistance nature [61]. OS stem-like cell model has been established for determining the effects of metformin in OS MG63 cells, where sphere assays is involved in formation of OS spheres with suppressive potential. The present practice of applying metformin not only is involved in inhibition of proliferation in both OS MG63 cells and OS stem-like cells, but also in inhibiting of stemness via targeting Oct-4 and Nanog [62].

### Signaling pathways and epigenetic regulators

Evolving evidence has provided appreciation of the role of many molecular signaling pathways for CSCs that may be activated or suppressed to play a key role in malignancies. Current evidence supporting pivotal role for many signaling pathways in OSl stem cells (e.g., Hedgehog, Wnt/β-Catenin, Notch, MAPK).

These signaling pathways have been found to conversely regulate the activity of the normal stem cell, where the properties of the CSCs are involved, in either the abnormal activation or suppression, including self-renewal, the formation of chromosomes, cell proliferation and differentiation as well as an aggressive nature, metastatic potential, and cancer-drug resistance.

It should be taken into consideration that these pathways have its own specific complexity and various regulatory factors are involved in regulation of the quiescent state of CSCs, including the extrinsic and intrinsic molecular signals; therefore, they form the interwoven networks of mediators that provide inter-pathway cross talk [17]. The role of the Notch, Wnt and Hedgehog signaling p.

On the other hand, combination of genetic events and epigenetic regulators has been previously revealed to be involved in abnormal cellular differentiation patterns during carcinogenesis, where current evidence supporting an important role for epigenetic regulators in modulating stems cell properties. Epigenetic modulating intervention has also been evaluated for targeted therapy of cancers [63]. Present evidence supporting a role for Nanog in maintaining OSCs, while imprinted gene TSSC3 is not only responsible for inhibition of stem-like phenotype, but also play a key role in repressing Nanog expression through inhibiting the Src/Akt pathway, suggesting that targeting TSSC3 and Nanog can be a new strategy for improving prognosis [64].

**Fig. 2:**
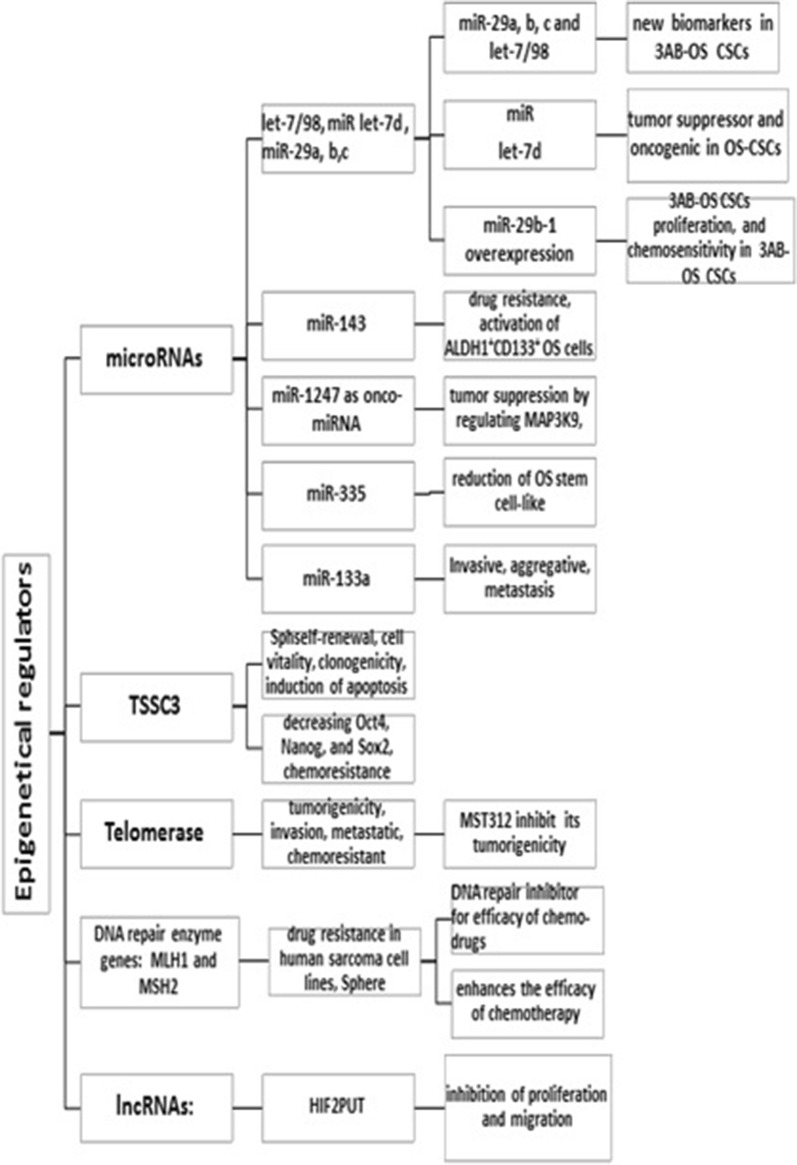
Master epigenetic regulators of bone sarcoma stem cells in a glance as described previously

Pathways and epigenetic regulators are highlighted in Figs. 1, 2 based upon the current evidence.

### The role of MAPK/ERK signaling pathway in OS stem-like cells

Role of MAPK/ERK signaling pathway has been previously highlighted in OS stem-like cells; However, its role in bone sarcomas is currently being untraveled, suggesting further investigations. As a matter of fact, ERK1/2 (p44/42 mitogen-activated protein kinases) has been revealed to play a potential role in tumorigenesis and stemness of 3AB-OS cells. This pathway has been shown to be implicated in the cytoskeleton rearrangement in tumoral cells invasion, indicating its association with increased motility and invasion of 3AB-OS stem-like cells as previously reported [65, 66]. The role of MAPK pathway needs to be a subject of future investigation for OSCs. Targeting MAPK/ERK1/2 may provide therapeutic effects and need to further investigation in experimental and clinical trial data [67].

#### Hedgehog

The HH pathway is considered of particular importance as a target for cancer therapeutic strategies, where the HH pathway in normal progenitor/stem cell renewal has been concisely addressed to play a key role in the regeneration of many organs including bone, prostate, liver, bladder, etc. [68–72]. It is worth noting that normal activity of the HH pathway is associated with tissue homeostasis and repair, while its uncontrolled activities contribute to cancer development [73]. The Hh signaling is involved in driving the CSC phenotype via regulation of ALDH, WNT2, and BMI1, as well as CD44 expressions, which its activation has been reveled in in CSCs [73, 74]. The Hh pathway is inappropriately involved in maintenance of CSC in different human cancers [73]. A number of the Hh signalling pathway inhibitors have employed to suppress the inappropriate activation of this pathway in clinical therapies of human cancer.

Hh signaling components are described as Indian Hh, Hh ligands, Desert Hh, GLI transcription factors (GLI1, GLI2 and GLI3), Smoothened (SMO) and Patched (PTCH; PTCH1 and PTCH2), which the two last core components were proximally found in 70% of OS [75]. Meanwhile, both ligand-dependent (co-expression of IHH-PTCH1) and independent inhibitors (SMO; PTCH1; GLI) may be considered as effective Hh signaling inhibitors in OS, while their effects has been shown in many cancers such as basal cell carcinoma and medulloblastoma [75, 76]. Hedgehog/Smoothened inhibitor (NVP-LDE-225) has been used for PI3K/mTOR inhibition, which leads to a decrease in CSC self-renewal capability, in pancreatic CSC [77], and Vismodegib (GDC-0449) have approved in 2012 with therapeutic benefit by FDA for metastatic or locally advanced BCC [78]. Thereafter, another SMO inhibitor, sonidegib was suggested as FDA-approved anti-HH pathway strategy for treatment of locally advanced BCC [79]. Current therapeutic strategies are now being explored SMO inhibitors for OS. However, none of them is favorably applied for the clinical treatment of OS [77]; nonetheless, further studies are required in terms of CSC inhibitor in OSCs. Targeting the Hh pathway is being explored to eradicate CSCs, which this pathway may provide a valid therapeutic target in OS due to its role in pathogenesis of OS [77, 80].

#### Targeting Wnt/β-catenin

The Wnt pathway is characterized by a protein family that play a pivotal role in many cellular mechanism such as organogenesis, cell survival and stem cell renewal. Extracellular Wnt is involved in intracellular signal transduction pathway in clouding canonical (Wnt/beta-catenin dependent) and non-canonical pathways (beta-catenin-independent), [81].

The role of the Wnt/beta-catenin pathway in tumorigenesis is under debate, while its osteogenic differentiation potential has been previously reported. On the other hand, its regulatory effects on cancer stem cells (CSC) have been revealed [82].

The transcriptional co-activator β-catenin plays its role in development of gene expressions; therefore, inhibition of Wnt/β-catenin signaling has been aimed to assess its potential effects for cancer therapy, a number of protein are capable of modulating the Wnt/β-catenin pathway, including sFRPs, WIF, DKK proteins (DKK 1,2,3), and sclerostin, as well as small molecules [83].

An increase in drugs sensitization was previously observed when WNT and NOTCH pathways were inhibited in OS cell lines [84]. It is worth noting that DKK1 (Dickkopf-related protein 1) as enhancer of protumorigenic features is able to suppress the canonical WNT pathway, where is linked to noncanonical JUN-mediated WNT pathways, and can consequently play a key role in mediating tumorigenic potential, partly, by ALDH1A1 and stress response enzyme overexpression. Aberrant Wnt/β-catenin signaling has been also recognized to be linked to overexpression of Sox-2, nestin Oct-4, CD133, and Nanog as stem cell proteins and tumorigenicity, supporting a role for Wnt/β-catenin signaling and its downstream pathway in eliminating OS-CSCs [85]. As indicated in Table 2, salinomycin is capable of inhibiting Wnt pathway activity via degradation of β-catenin as an inhibitor of OS stem cells, indicating the role of Wnt/β-catenin signaling [86]. JW74 (Tankyrases1, 2 inhibitors) exhibited an important role in inhibiting Wnt/β-catenin signaling, where tankyrase play a role in cell cycle progression, reduction of differentiation and apoptosis in OS cell lines [87]. However, gastrointestinal toxicity is a concern in terms of these inhibitors, where further investigations are recommended [88]. Contrary, some studies reported inactivation of Wnt/β-catenin signaling in OS specimen and cell lines [89, 90]. The Wnt pathway plays an important role in bone formation and agonists, this effects is reduced due to single bisphosphonate doses [81, 91].

**Table 2:** Pre-clinical development for OSCs-targeting agents

Therapeutic	Function of molecules	Study type and model	Mechanism	References
BYL719	A new specific PI3Kα inhibitor	Different murine preclinical models of osteosarcoma	Tumor development and tumor ectopic bone formation by decreasing Ki67+ cells and tumor vascularization	Gobin et al. [53]
LY294002	An inhibitor of phosphoinositide 3-kinases (PI3Ks)	Human osteosarcoma CSCs, in vitro study	Prevent phosphorylation of PKB/Akt via inhibition of PI3K phosphorylation activity, leading to G0/G1 cell cycle arrest and apoptosis	Gong et al. [108]
BRM270 as a compound from 7 seven sian medicinal plants	NF-κB inhibitor	An in vivo tumor metastasis model of nude mice for osteosarcoma	A NF-κB inhibitor via acting as a suppressor of NF-κB signaling cascade in multidrug resistance -induced stem like cancer-initiating cells	Kwon et al. [194]
parthenolide	NF-κB inhibitor and oxidative stress inducer	LM7 osteosarcoma cells, in vitro treatments	Parthenolide and ionizing radiation leads cell death in osteosarcoma cells, resulting in reduction in the viability of both the osteosarcoma cells and CD133^+^ CSCs	Zuch et al. [110]
MC1742 and MC2625	HDAC inhibitors	CSCs, in vitro study	Can elevate acetyl-H3 and acetyl-tubulin levels, inhibited CSC sphere growth via apoptosis induction in osteosarcoma and Ewing sarcoma	Di Pompo et al. [195]
Bufalin	–	In vitro study, and in vivo (nude mice model)	Inhibits the differentiation and proliferation of CSCs from C1OS via targeting miR-148 Downregulate proliferation marker Ki67, resulting in inhibition of sphere forming and proliferation in human OSCs derived from the MG63 cell line	Chang et al. [182, 183]
Salinomycin	Antibacterial and coccidiostat agent	Both in vitro and in vivo study (nude mice model)	Suppress the sarcosphere formation, expressions of Oct4 and Sox2. Inhibit Wnt/β-catenin pathway via degradation of β-catenin and can act as inhibitor of OSCs	Tang et al. [86]
CESP^a^	Nanoparticle	Both in vitro and in vivo study (mic model)	Effective inhibitor of tumor growth with promising efficacy in osteosarcoma-bearing mice, effectively target CSCs of osteosarcoma and cancer cells	Chen et al. [196]
Ap-SAL-NP^b^	Nanoparticle	Both in vitro and in vivo study (mic model)	Ap-SAL-NP kill CD133^+^ osteosarcoma CSCs	Ni et al. [197]
EGFR-SNPs	Nanoparticle	In vitro drug assessment	EGFR-SNPs decrease the osteosarcoma sphere-forming and CD133^+^ osteosarcoma CSCs while comparing with salinomycin and SNPs. Effectively enhance delivery of salinomycin to osteosarcoma cells	Yu et al. [198]
5-Azacytidine	DNA methyltransferase (DNMT) inhibitor	Osteosarcoma cell lines Saos-2 and MG63	Induce an increase of stemness features of OS cells in context of CD133, Sox2, OCT4 and Nanog overexpression, as well as high sarcospheres-forming efficiency	Tirino et al. [199]

^a^Sali-entrapped lipid-polymer nanoparticles conjugated with EGFR and CD133 aptamers

^b^Salinomycin-loaded PEGylated poly nanoparticles conjugated with CD133 aptamer

As aforementioned, this pathway plays a pivotal role in many cellular mechanisms such as organogenesis, cell survival and stem cell renewal [92]. In this regard, concerns have risen, which inhibition of the Wnt pathway may result in a negative effect on the normal Wnt-dependent stem cell in many aspects such as gastrointestinal tract and fast turnover (e.g., hair follicles), Furthermore, cross talk between the cell signaling pathways should be considered in therapeutic strategies [81].

#### Notch

The notch signaling pathway can be activated by ligand binding to Notch receptors. Mammals possess four receptors, Notches (1–4) and five ligands, Delta-like (DLL1, DLL3, and DLL4) and the Jagged1–2 [93, 94]. Activation of the notch signaling pathway lead to the cancer metastasis and its role in the relationship between CSCs self-renewal and angiogenesis have attracted a therapeutic targeting of Notch signaling in eliminating CSCs. The inhibition of this pathway is considered as an emerging therapeutic target for cancer by eradicating the CSCs [95].

As studies indicated, notch pathway was not uniformly expressed across a tumor and OS cells are likely to exhibit low levels of Notch ligand expression except regions adjacent to blood vessels [96]. However, it has been reported that metastatic OS cell lines exhibited an increased levels of NOTCH receptors, Delta like canonical Notch ligand 1 and enhancer of split-1 (HES-1), indicating the potential role of NOTCH signaling in increasing the OS metastasis [97]. Moreover, Notch pathway manipulation may lead to different cell-autonomous behaviors among cell lines.

A large number of inhibitors have been suggested based upon preclinical studies, especially consisted of gamma secretase inhibitors (GSI), antibody targeting Notch receptors or ligands and siRNA [94, 98]. Most therapeutic strategies have revealed γ-secretase -targeting cancer therapies, which have been clinically applied GSI. It has been demonstrated that GSI is capable to remarkably represses CSCs) as suggested by in vitro studies, [99].

Additionally, gamma secretase enzyme complex has a number of other protein targets that can be involved in OS behavior, including CD44, Her-4, and the WNT/β-catenin signaling [96]. WNT and Notch pathways have been obviously demonstrated to participate in the OS development [10, 94, 100]. The inhibitory role of GSIs has been demonstrated to be remarkable in CD133+ cells, indicated that inhibition of the Notch pathway can be potentially considered as a strategy for targeting cancer stem cells [94, 101, 102]. The combination of GSIs with other therapeutic strategies such as ionizing radiation, chemotherapy drug and signal transducers has been previously evaluated [103–105]. Current evidence reveals that the pharmacological inhibition of the Notch pathway is a potential therapeutic strategy for overcoming chemoresistance, where Notch inhibitors exhibited synergistic effect, showing their role in improving chemotherapy response [94].

Current evidence has suggested an oncogenic role for Notch in OS, where this pathway was found to be related to ALDH expression, and metastasis. There is evidence of regulatory properties for Notch when have a strong impact on ALDH activity, and its up-regulation was linked to overexpression of ALDH. In a murine OS cells, it has been revealed that inhibition of Notch signaling was linked to ALDH activity and the metastatic phenotype. Notch and ALDH can participate in OSC maintenance, chemoresistance, and metastatic potential [106], suggesting that their therapeutic potential as putative targets.

#### PI3K signaling

PI3K signaling was being investigated for its oncogenic potential and maintenance of CSCs, where PI3K inhibitors may be involved in inhibition of CSCs. PI3K-targeting studies has described its role in clinical trials for cancer therapeutic approach [107]. LY294002 has been exhibited inhibitory effect on phosphorylation of PKB/Akt through its preventive role in the PI3K phosphorylation, leading to cell cycle arrest, and apoptosis in OSCs, indicating key role of PI3K/Akt pathway. Current evidence supports the contribution of PI3K inhibitors for controlling OS through targeting CSCs [53]. BYL719 was being shown to be a favorable drug, where exhibited its important role at inhibiting cell migration and inducing cell cycle arrest in OS cells [108]. VS5584as an inhibitor of mTORC1/2 was strongly exhibited inhibitory role in the growth and survival of CSCs, when comparing with non-stem-like cancer cells, meanwhile VS5584 was favorably targeted CSCs [109].

#### NF-κB signaling pathway

Activation of nuclear factor κB (NF-κB) has been demonstrated in radioresistant subpopulations of OS cell lines; whereas parthenolide proved to facilitate sensitization of mentioned subpopulations to radiation and markedly is based on its inhibitory function [110]. Furthermore, BRM270 has been demonstrate to decrease tumorigenic potential via suppression of NF-κB signaling in multidrug resistant OS stem-like cells, where targeting of NF-κB, and Cdk6 with IL-6 have provided support for programmed cell death and development of drug resistance therapy for CSCs [111]. Further preclinical and clinical trials are needed to clarify the potential of NF-κB signaling.

#### SDF-1 (cxcl12) cxcr4

The SDF-1α/CXCR4 signaling pathway involved not only in tumor cell proliferation, migration and angiogenesis, but also in immune surveillance of tissues [112, 113]. CXCL12 is expressed in both MSCs and osteoblasts in bone marrow [114], and revealed to play a key role in facilitating entrance of CSCs expressing CXCR4 into the bone microenvironment. On the other hand, CXCR4 expression has been found to be involved in maintaining CSCs’ stemness, while CXCL12 was found to play an important role in attraction of CSCs to the bone marrow niches [115]. CXCR4 receptor of CXCL12 has been revealed to be overexpressed in in the BME, and OSCs [23].

Increasing evidence demonstrates that the SDF-1/CXCR4 signaling pathway is not only responsible for the hematopoietic stem cell maintenance, but also play a key role in metastatic processes, indicating a potential role this pathway in the of OSCS subpopulations evolution [116]. Targeting SDF-1 and neutralizing CXCR4 represented a therapeutic strategy for cancer, both of which depicted a elevated expression level in many kinds of tumor cells [117–119]. Therefore, molecule inhibitors targeting the SDF-1 CXCR4 signaling are considered in cancer therapy.

### Targeting the tumor microenvironment of CSCs

**Fig. 3:**
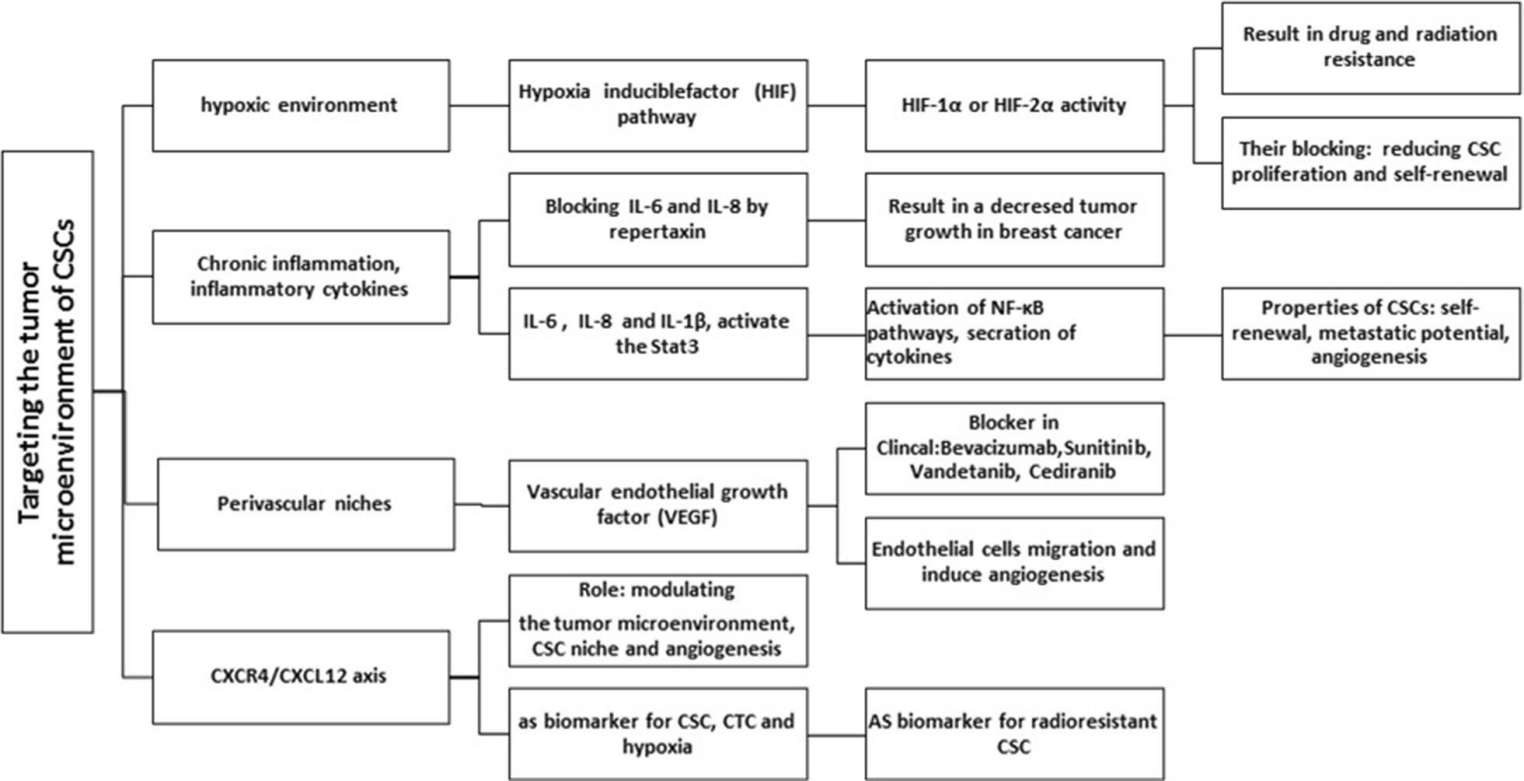
Many types of stem cell microenvironments of CSCs are summarized in a glance

OS has shown heterogeneity with various mutations in the genes that are generated by chromothripsis [120]. It has been revealed that the micro-environment of the tumor may play an important role in regulating this phenomena via molecular mechanisms, where needed in-depth understanding (Fig. 3). Based on the current evidence, it has been revealed that oxygen tension and microenvironment are implicated in developing cancer [121, 122].

Hypoxia has been described to be a major feature of the micro-environment of the tumor that has been recognized to play a key role in increasing tumor growth and indicated as a contributor to the CSC phenotype [123]. The current evidence suggests the regulatory role of hypoxia under the CSC population and maintaining the normal tissue in a stem cell states [122, 124, 125]. Furthermore, hypoxic areas of the tumor is likely to act as a niches for SCS, and hypoxic environment can provoke cellular reprogramming for generating IPS colonies [121, 125].

The expression level of hypoxia-related gene has been linked to the activation of hypoxia-inducible factor (HIF) and HIF-1α and HIF-2α [126–128]. HIF pathway (HIF-1α or HIF-2α activity) has been revealed to be associated with promotion of cancer cell stemness, where blocking HIF-1α or HIF-2α activity has been strongly contributed to self-renewal and proliferation capacities [4, 121, 129].

TGF-β1 signaling and a hypoxic environment has been demonstrated to contribute to the provoking self-renewal capacity in non-stem OS cells, where this event not only promotes tumorigenicity, metastatic, neovasculogenesis, but is also linked to drug resistance characteristics [61].

The acidic micro-environment around the hypoxic cells in combination with the activation of a group of proteases potentiates metastasis. Hypoxic cells are described to be less likely to accumulate the therapeutic concentrations of anti-cancer drugs, which result in multiple drug resistance, due to their undesirable angiogenesis and the inaccessibility of their locations [123]. It is worth noting that targeting the CSC niche hypoxia with chemotherapy may be a future strategy against CSCs based on the recent report, hypoxia and stem cell mediated therapy can be crucial in removing the CSC, which will bring therapeutic strategies for bone tumors [122]. Hematopoietic stem cells (HSCs) have been previously found to be at the lowest level of an oxygen gradient, indicating the regulatory role of hypoxia for stem cells function in the bone marrow [130].

The CXCR4–CXCL12 axis has been demonstrated to play a key role in cancer-cell-tumor microenvironment interactions, whereas is known as metastasis-associated marker for bone [114, 131].

CXCL12 has been found to be expressed by both MSCs and osteoblasts in bone marrow [115], and play a key role in facilitating entrance of CSCs expressing CXCR4 into the bone microenvironment. CXCR4 expression is linked to maintaining CSCs’ stemness, while CXCL12 is contributed to attraction of CSCs to the bone marrow niches [114]. However, further investigations are needed for clarifying the role CXCR4–CXCL12 axis in OSCs.

Future progress can be helpful in deep understanding of biology involved in the native stem cell niches, where various mechanisms and strategies are applied by niche components for supporting stem cell function. On the other hand, it is noteworthy that there is not much information about microenvironment of OSCs. However, it should be taken into account that the micro-environment is involved in the development of OSCs and affects its biological behavior, Nevertheless, the exact understanding of molecular interactions, microvessels and hypoxia would be very effective in targeting novel therapies for OSCs.

### Molecular imaging

#### Circulating tumor cells

Tumor cells (CTCs) isolated from the blood stream of patients suffering from localised to metastatic cancers may have prognostic significance. It is worth noting that CSCs and or circulating tumor stem cells (CTSCs) are also recently considered as a small subset of CTCs. Nevertheless, further studies in the future are needed not only for detecting CTCs, but also for characterizing those [132, 133]. A growing body of evidence suggests a significant association between CSCs and CTCs, where revealed various functional states of the same related subset of cancer cells [134, 135]. Cancer cells in the peripheral blood has been found to be linked to disseminated disease and an increased risk of tumor progression; where, increasing evidence indicated the clinical use of CTCs detection for prognosis and follow-up of patients suffering from many kinds of cancers [132, 136–139]. However, prognostic significance of CTCs has not yet been clarified in patients with early-stage diseases without metastasis [132].

CTCs are a heterogeneous and rare population of cells in the blood, which despite the tremendous advances in recent years, have not been adequately characterized because of their heterogeneity and dynamism [140, 141]. The mechanisms that release the CTCs from the tumor are not completely determined It is not known, therefore, whether the CTCs represent only represent a subset of cancer cells makeup in the tumor, or are involved in entire [142]. A question that requires more studies to be clarified.

The development of new methods for efficient detection and characterization of CTCs, especially CTSCs, in peripheral blood samples can provide a basis for improving patient survival by targeting of these cells, where many methods are provided for solation and detection of CTCs based on the use of enrichment and detection steps. Nevertheless, substantial variability has been achieved for CTC data based upon the use of different methods for detecting CTC [143].

Studies are assessing CTC-directed therapies to improve treatment outcomes and to reduce CTC numbers or even eliminate CTCs in response to treatment, where these may be likely linked to long-term survival [141, 144]. There is no specific marker for the isolation of CTC sarcomas, using a preclinical model reflecting human OS [145–149]. Nonetheless, increasing evidence has gradually led to an increase in studies on the biological role of CTCs in OS, where an increased number of CTC has been found in metastatic patients [150]. A growing body of evidence suggested clinical significance of CTCs in small cohorts of OS, where a positive correlation has been found between CTCs numbers, poor prognosis and disease progression by applying FISH method [151, 152]. Chalopin et al. suggested a detectable number of CTCs in blood circulation at primary stage of OS, while paradoxal effect of ifosfamide have revealed among subjects recorded as displaying evidence of therapeutic response on established/para-osteal tumors; however no impact was observed on sub-clinical disease [145].

Overall, it should be taken into consideration that a reduction in the CTC does not adequately provide the exacted insight into the biology of the tumor and therapeutic responses, whereas rare CTSCs remained unaffected, indicating the importance of identifying CTSCs for providing treatment strategies [141, 153]. CTCs may be only linked to tumor burden or represent the leakiness of tumor-related vasculature [142, 154].

#### Circulating DNA

As indicated, tumor cell releases two kinds of material in the blood stream including CTCs and cell-free circulating tumor DNA (ctDNA), [155]. CTDNAs as blood-based biomarkers are (liquid biopsy biomarkers) are available for cancer diagnosis, prognostic determination, and effective treatment in the early stages [156–158]. Tumor-related genetic and epigenetic alterations are the basis for identification and tracking ctDNAs. However, more validation is required for ctDNA before their widespread clinical trials. It is worth noting that CtDNA not only spreads into the bloodstream via apoptosis or necrosis of the CTCs, but also their release can be originated from the primary tumor or metastatic lesions [159].

They consisted of 160–180 bp, indicating the degradation of DNA to nucleosomal units that is considered as properties of the apoptotic process [160, 161]. It has been revealed that CTCs has less sensitivity for detection of tumor related genetic rearrangements in comparison with ctDNA, due to low detectable number of CTCs in blood circulation [157]. Determining tumor proliferation and metastases in CTCs are performed using DNA methylation [162]. Furthermore, ctDNA technologies provide a series of analyses including single gene mutational evaluation, next generation deep genome sequencing, following analysis of methylation, where their application covers all stages of cancer management [163].

The ctDNA analysis as liquid biopsy provides an opportunity to accurately assess the status of cancer patients with a much cheaper, faster, and reliable method, because ctDNA serve as promising diagnostic, prognostic, and predictive marker. In addition to commonly used DNA recognition methods, including PCR, real-time PCR, Digital PCR based techniques (e.g., droplet digital PCR), PARE and BEAMing, [157, 164, 165]. Recent developments in Next Generation Sequencing (NGS) provide a new method for ctDNA investigation. CtDNA can also be used as a medical tool available to physicians for changing current approach in personalized cancer management, since it is capable of providing accurate data about each patient. However, this technique still needs further optimization [157, 164].

Nevertheless, ctDNA investigation should be considered in light of some limitations. Its sensitivity for early detection can be limited by low amount of ctDNA because of low tumor burden, leading to 0.02% MAF sensitivity. A number of advanced technologies can facilitate methods required for enhancement of the sensitivity including unique molecular identifiers (UMIs) in the amplification step preceding sequencing and the in vitro and in silico improvement of ctDNA using virtue of shorter fragment length of them [163].

The use of ctDNA can be of particular benefit as a liquid biopsy because is capable of capturing full complex OS heterogeneity and tracking genomic evolution [166, 167]. Somatic mutations have been previously detected in cell-free DNA of patients suffering from OS by comparing tumor-germline pairs [168]. Furthermore, NGS has been applied in a study composed primarily of tumor sequencing findings capable of detecting ctDNA in half of the plasma specimen from subjects with OS, where its detection was found to be strongly linked to inferior outcomes [169]. Ultra-low-pass whole-genome sequencing (ULP-WGS) as a NGS method is used nowadays for detection of the complex structural variants among OS patients [170]. Moreover, translocation related vs. complex structural variants are currently being used in pediatric malignancies, which are improved study by providing the reliable detections of ctDNAs [169].

Two specific markers EpCAM and cytokeratins have been approved using cell search (Veridex) system for CTCs of epithelial origin, but there have not been markers for sarcomas. It is worth noting that the cell search system was not capable of detecting sarcoma-derived CTCs, while other method has been provided for identifying them without OS CTCs [152, 171, 172].

### An overview of therapeutic strategies for targeting OSCs

CSCs form a small fraction of the tumor cell population, possibly combinations of anti-CSC agents and debulking therapeutic approaches can be successfully applied in clinical trials [50]. Targeted therapeutic strategies are remarkably considered to be more specific as compared to conventional chemotherapeutic strategies. The clarification of sophisticated molecular mechanisms has led to the design of targeted therapies that provide genomic and transcriptional data on specific gene regulated during tumorigenesis, leading to deep design of therapeutic options for many kinds of cancers [173]. Nonetheless, there are little agents that have been approved for targeting bone sarcoma stem cells in clinical trials, but different compounds has been suggested as candidates in CSCs clinical trials.

We discussed potential targets of OSCs; hence, it is required to mention the potential drugs that have been applied in clinical trial or only showed effects in animal models. Therefore, a detailed Table is summarized that would be helpful (Tables 2, 3, 4), which more information are provided regarding therapies, such as kind of inhibitors (chemical or biological).

**Table 3:** Inhibitor used as new therapeutic approaches for osteosarcoma in clinical trails (Multitarget inhibitors, IGF1-R inhibitors, mTOR inhibitors and inhibitor of Wnt/β-catenin signaling)

Status	Conditions	Interventions	Phase	Last update posted
Unknown	Osteosarcoma	Drug: Chemotherapy Drug: Endostar (drug type: VEGFR inhibitor)	Phase 2	September 3, 2014
Completed	Osteosarcoma	Drug: sorafenib^b^ (multi target inhibitor: PRGFR/VEGFR inhibitor)	Phase 2	March 28, 2013
Recruiting	Soft tissue sarcoma Bone sarcoma	Sunitinib [Sutent] (multitarget inhibitors: (e.i.,PDGF-Rs, VEGFRs)	Phase 1	September 11, 2017
	Metastatic osteosarcoma Relapsed osteosarcoma	Drug: Sorafenib^b^ (multitarget inhibitors; PRGFR/VEGFR inhibitor) Drug: Everolimus (mTOR inhibitor)	Phase 2	June 17, 2015
Completed	Sarcoma	Drug: RG1507 (IGF1-R inhibitors)	Phase 2	April 4, 2017
Completed	Sarcoma	Drug: RG1507(IGF1-R inhibitors)	Phase 2	April 4, 2017
Unknown	Osteosarcoma	Dietary supplement: Curcumin powder (WNT/β-catenin inhibitor) Dietary Supplement: Ashwagandha extract	Phase 1 Phase 2	June 23, 2011
Completed	Osteosarcoma	Drug: Saracatinib a Src inhibitor of c-Src) Drug: Placebo	Phase 2	May 11, 2018
Terminated	Osteosarcoma Metastatic osteosarcoma	Drug: Pazopanib^c^ (drug type: multitarget inhibitors: VEGFR inhibitor)	Phase 2	June 26, 2018
Unknown	Refractory or relapsed osteosarcoma	Drug: Everolimus (mTOR inhibitor)	Phase 2	August 7, 2013
Completed	Osteosarcoma Metastasis	Drug: Apatinib (YN968D1, tyrosine kinase inhibitor and VEGFR2 inhibitor)	Phase 2 Phase 3	April 23, 2018
Active, not recruiting	Osteosarcoma Malignant fibrous histiocytoma (MFH) of bone	Biological: Bevacizumab (drug type: monoclonal antibody; target: VEGF-A) Drug: Cisplatin Drug: Doxorubicin (and 5 more…)	Phase 2	June 14, 2018
Completed	Sarcoma	Biological: trastuzumab (drug type: monoclonal antibody; target: ERBB2) Procedure: conventional surgery	Phase 2	June 21, 2013
Terminated	Osteosarcoma Sarcoma, Ewing’s Peripheral neuroectodermal tumor	Biological: Robatumumab (SCH 717454; IGF1-R inhibitors )	Phase 2	June 7, 2017
Recruiting	Metastatic Ewing sarcoma Metastatic osteosarcoma Recurrent Ewing sarcoma (and 7 more…)	Drug: Cabozantinib S-malate (small molecule receptor tyrosine kinase (RTK) inhibitor)	Phase 2	May 24, 2018
Recruiting	Ewing sarcomas Chondrosarcomas Osteosarcomas Chondroma	Drug: Regorafenib (multi-kinase inhibitor Drug: Placebo	Phase 2	August 16, 2018
Completed	Metastatic soft-tissue sarcomas Metastatic bone sarcomas	Drug: Ridaforolimus (mTOR inhibitor) Drug: Placebo	Phase 3	February 13, 2015
Recruiting	Adult liposarcoma Metastatic liposarcoma Metastatic osteosarcoma (and 4 more…)	Drug: Pazopanib^c^ hydrochloride (multitarget inhibitors; target: PDGFR, c-Kit,) Drug: Oral Topotecan hydrochloride	Phase 2	January 9, 2018
Completed has results	Metastatic osteosarcoma Recurrent adult soft tissue sarcoma Recurrent osteosarcoma (and 2 more…)	Biological: Cixutumumab ( drug type: monoclonal antibody; target: IGF1R) Drug: Temsirolimus	Phase 2	July 30, 2015
Completed	Childhood malignant fibrous histiocytoma of bone Sarcoma	Drug: Imatinib^a^ mesylate (PDGFR inhibitor)	Phase 2	June 19, 2013
Completed	Glioblastoma Rhabdomyosarcomas Neuroblastoma Osteosarcoma	Drug: Irinotecan (Camptosar), Gefitinib (ZD1839: Drug type: EGFR inhibitor )or (Iressa)	Phase 1	April 17, 2012
Terminated has results	Sarcoma	Drug: Sorafenib^b^ (PRGFR/VEGFR inhibitor) Drug: Ifosfamide	Phase 2	November 24, 2015
Recruiting	Liposarcoma Osteogenic sarcoma Ewing/Ewing-like sarcoma Rhabdomyosarcoma	Drug: Regorafenib Drug: Placebo	Phase 2	July 18, 2018
Completed	Leiomyosarcoma Liposarcoma Osteosarcoma (and 2 more…)	Drug: Ridaforolimus (mTOR inhibitor)	Phase 2	February 13, 2015
Completed	Sarcoma	Drug: RG1507 (drug type: monoclonal antibody; Target: IGF1R )	Phase 2	April 4, 2017
Withdrawn	Sarcoma Neuroblastoma Wilms tumor (and 2 more…)	Drug: Pazopanib^c^ (drug type: VEGFR inhibitor), (GW786034)	Phase 1	July 2, 2017
Completed has results	Metastatic Ewing sarcoma/peripheral primitive neuroectodermal tumor Metastatic osteosarcoma Recurrent adult soft tissue sarcoma (and 6 more…)	Drug: Sorafenib^b^ tosylate (multitarget inhibitors: PRGFR/VEGFR inhibitor) Procedure: Therapeutic conventional surgery Other: Laboratory biomarker analysis (and 3 more…)	Phase 2	April 30, 2014
Recruiting	Glioma Rhabdomyosarcoma Osteosarcoma (and 5 more…)	Biological: Erlotinib (drug type: PDGFR Inhibitor) Drug: Temozolomide	Phase 2	February 15, 2018

IGF1-R pathway; Targeting mTOR pathway as a downstream pathway; Multi-target inhibitors; Wnt/β-catenin signaling

^a^Acts as inhibitor of PDGFR, BCR-ABL and c-KIT

^b^Acts as inhibitor of VEGFR, PDGFR, RET. BRaf, and c-KIT

^c^Acts as inhibitor of VEGFR1-3, PDGFRα/β, c-KIT

**Table 4:** New therapeutic agents used for osteosarcoma by targeting osteoclasts and immune system based on the clinical trials (Niche cells and their signalling), data provided from https://clinicaltrials.gov

Status	Conditions	Interventions	Phase	Last update posted
Recruiting	Osteosarcoma	Drug: Avelumab (monoclonal antibody)	Phase 2	June 25, 2018
Recruiting	Osteosarcoma	Drug: Pembrolizumab (IgG4 isotype antibody)	Phase 2	April 5, 2018
Recruiting	Metastatic osteosarcoma	Drug: Sm-EDTMP Other: Autologous stem cell infusion Radiation: External beam radiotherapy	Phase 2	February 14, 2018
Unknown	Osteosarcoma	Drug: Zoledronic acid (type of drug: bisphosphonates; target: Osteoclasts) Drug: Standard chemotherapy	Phase 2 Phase 3	June 23, 2011
Active, not recruiting	Recurrent osteosarcoma	Drug: Glembatumumab Vedotin Antibody–drug conjugate Other: Laboratory biomarker analysis Other: Pharmacological study	Phase 2	May 7, 2018
Completed	Osteosarcoma	Drug: Cisplatin Drug: Doxorubicin Drug: Methotrexate Administration of Pamidronate (drug type: bisphosphonates; target: osteoclasts) with chemotherapy	Phase 2	January 20, 2016
Recruiting	Recurrent osteosarcoma	Biological: Humanized anti-GD2 antibody (drug type: monoclonal antibody; target: immune system) Drug: GM-CSF	Phase 2	August 2, 2018
Active, not recruiting	Childhood osteosarcoma Metastatic osteosarcoma Recurrent osteosarcoma (and 3 more…)	Biological: Denosumab (drug type: monoclonal antibody; target: RANKL)	Phase 2	August 7, 2018
Completed	Sarcoma	Zoledronic acid (type of drug: bisphosphonates; target: osteoclasts Drug: cisplatin Drug: Dexrazoxane hydrochloride Drug: doxorubicin hydrochloride (and 10 more…)	Phase 1	July 4, 2014
Completed	Neuroblastoma Melanoma Osteosarcoma Ewing sarcoma	Biological: Anti-GD2 antibody (drug type: monoclonal antibody; target: immune system)	Phase 1	
Terminated	Sarcoma	Zoledronic acid (type of drug: bisphosphonates; target: osteoclasts Drug: Cisplatin Drug: Doxorubicin hydrochloride Drug: Etoposide (and 4 more…)	Phase 3	June 22, 2016
Recruiting	Desmoplastic small round cell tumor Disseminated neuroblastoma Metastatic osteosarcoma (and 2 more…)	Biological: IL-2 Biological: GD2Bi-aATC (drug type: cells Biological: GM-CSF Evaluations of immune responses	Phase 1 Phase 2	December 4, 2017
Completed	Osteosarcoma	Drug: c (type of target: Monocyte/macrophage activator glycopeptide) Drug: Ifosfamide	Phase 2	March 22, 2017
Not yet recruiting	Bone sarcoma Soft tissue sarcoma	Biological: NY-ESO-1 (target: immunotherapy; TCR affinity enhancing specific T cell therapy)	Phase 2	March 16, 2018
	Ewing sarcoma Osteosarcoma Rhabdomyosarcoma	Biological: Expanded, activated NK cells	Phase 1 Phase 2	November 6, 2017
Active, not recruiting	Soft tissue sarcoma Bone sarcoma	Drug: Pembrolizumab: MK-3475 (cancer immunotherapy: target: PD-1 )	Phase 2	July 25, 2018
Recruiting	Recurrent malignant solid neoplasm Recurrent osteosarcoma Refractory malignant solid neoplasm Refractory osteosarcoma	Biological: Anti-SEMA4D monoclonal antibody VX15/2503	Phase 1 Phase 2	August 9, 2018
Recruiting	Ewing sarcoma Osteosarcoma Rhabdomyosarcoma	Biological: Expanded, activated NK cells (target: Immune system)	Phase 1 Phase 2	November 6, 2017
Completed	Sarcoma Osteosarcoma Neuroblastoma Melanoma	Biological: Anti-GD2-CAR engineered T cells Drug: AP1903 Drug: Cyclophosphamide	Phase 1	July 6, 2018
Recruiting	Ewing sarcoma Neuroblastoma Rhabdomyosarcoma (and 2 more…)	Procedure: Allogeneic HCT Drug: Donor NK Cell Infusion (target: immune system)	Phase 2	June 1, 2018
Active, not recruiting	Neuroblastoma Ewing sarcoma Rhabdomyosarcoma (and 2 more…)	Procedure: haploidentical stem cell transplantation and NK cell therapy (target: Immune system)	Phase 2	October 18, 2016
Recruiting	Neuroblastoma Rhabdomyosarcoma Osteosarcoma (and 3 more…)	Drug: Enoblituzumab (monoclonal antibody)	Phase 1	August 7, 2018
Not yet recruiting	Soft tissue sarcoma Bone sarcoma Chondrosarcoma (and 5 more…)	Drug: Ipilimumab (Drug type: monoclonal antibody; target: CTLA-4 receptor) Drug: Nivolumab (IgG4 anti-PD-1 monoclonal antibody; target: PD-1	Phase 2	November 1, 2017

Based on the data available on clinicalTrials.gov, many inhibitors including multitarget inhibitors, IGF1-R inhibitors, mTOR inhibitors and inhibitor of Wnt/β-catenin signaling have been applied to clinical trials (Table 3), where targeting of IGF1-R pathway, mTOR pathway as a downstream pathway, and Wnt/β-catenin signaling, as well as niche cells and their signaling (targeting osteoclasts and immune system) have been addressed in clinical trials and will provide a way to combine these targeting properties of the cells and their environment. Multitarget drugs have advantages of nti-angiogenesis therapy on OS (mostly with kinase activity); accordingly, many drugs such as Gefitinib, Everolimus Cixutumumab, R1507, Sunitinib, Pazopanib, Sorafenib, Bevacizumab have shown promise for OS based upon anti-angiogenesis therapy in clinical trial, while many clinical trials are evaluating therapeutic potential of angiogenesis inhibitors in many kinds of cancers (Table 3). The data of these agents is available online via https://clinicaltrials.gov/ct2/results?cond=OS&draw=6&rank=42#rowId41. Moreover, immune-based therapies such as Anti-GD2, GD2Bi-aATC, and mifamurtide, as well as stem cells and natural killer cells are potentially hampered cancer cells growth through harnessing of immune responses against tumors (Table 4), [174–176]. In addition, a number of bisphosphonates (Zoledronic acid and Pamidronate) have progressed to clinical trials for assessing their potential role in OS as simultaneous administration to chemotherapy (Table 4; ClinicalTrials.gov Identifier: NCT00691236; NCT00586846).

On the other hand, we listed a number of agents (inhibitors) in pre-clinical phases of development for which there has been key role in OS as OSCs-targeting agents (Table 2), such as PI3K, NF-κB, HDAC and DNMT inhibitors.

**Fig. 4:**
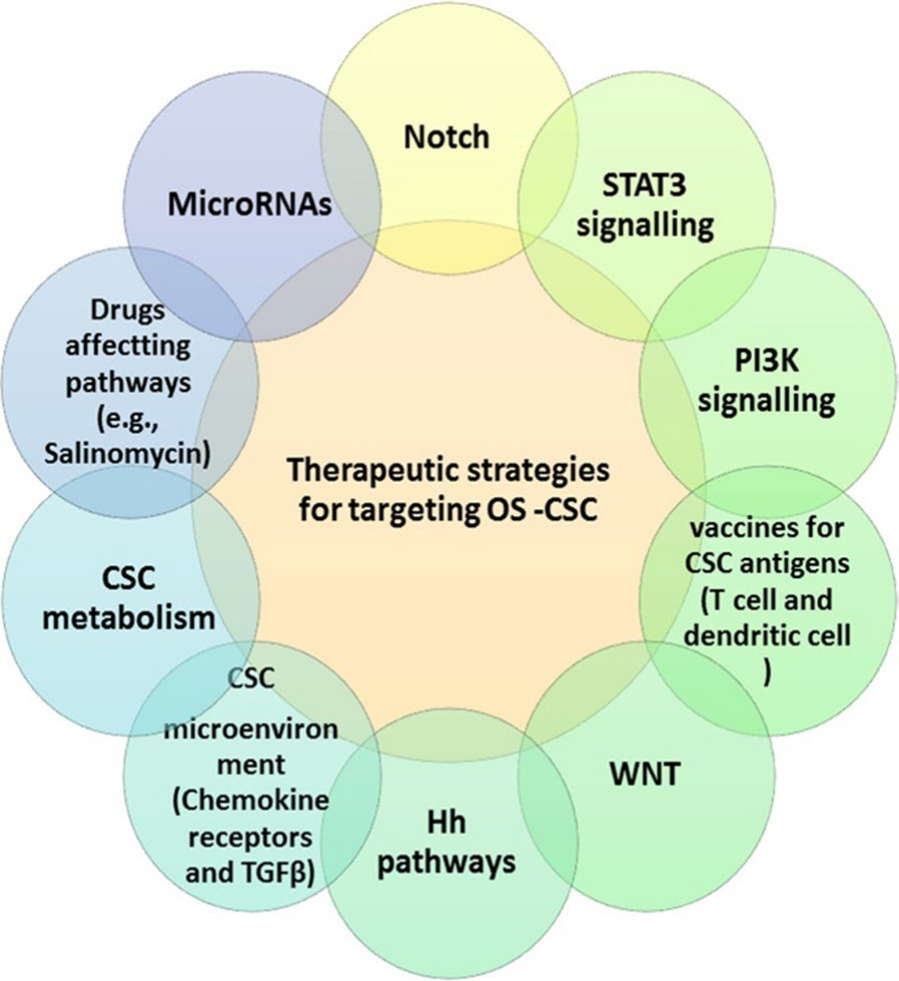
Therapeutic strategies for targeting OS-*CSC*

Overall, many therapeutic strategies have been suggested to control the CSC-related pathways (Fig. 4). Meanwhile, many pathways such as Wnt, Hedgehog, Notch, PI3K/AKT, TGFβ, and STAT3 are considered for therapeutic targeting by applying small molecules, antibodies and/or combinations of the two approaches [50, 52, 77, 177–179].

Notch, Wnt and Hedgehog pathways are potentially considered for developing immunotherapies and microRNA-mediated pathway inhibitors. Selective molecules inhibit mentioned signaling pathways via immune-based strategies by targeting several antigenic molecules of tumors, which consequently play their role by removing cancer cells via the use of innate immune responses [50, 178–180]. In terms of OS, many therapeutic approaches have been targeted receptor tyrosine kinases-mediated signaling WNT/β-catenin, as well as the mammalian target of rapamycin (mTOR), [10, 42, 181].

In light of this new evidence, cancer therapeutic strategies should not be limited to a single molecule or pathway. Therefore, it seems that a combination of OSCs-targeting agents with chemoradiotherapy, surgery, or immunotherapy, and/or targeting the tumor microenvironment of CSCs, will provide a much more potentially effective treatment in inhibiting tumor growth [50].

### Targeting CSCs by using drugs

#### Bufalin

Bufalin as a traditional Chinese medicine has been indicated to play a crucial role in suppressing differentiation and proliferation of OS cell line hMG63-derived CSC [182].

A previous study indicated the crucial role of bufalin in the miR148a and DNMT1 pathway for OS and miR-148a has revealed its role as a target of bufalin, where also showed its regulatory function for DNMT1 and p27 in mediating OS cells stemness. This aforementioned study also demonstrated inhibitory role of bufalin by inhibiting proliferation and differentiation of CSC [183]. However, it is worth noting that this therapeutic strategy need to further evaluation in clinical trial.

#### Salinomycin

Salinomycin has been described to be an antibiotic, which is implicated in cellular potassium flux, and show an anti- CSCs role [50, 184]. Current data support an effective role for Salinomycin in the inhibition of OS by targeting its stem cells. On the other hand, Salinomycin may be inhibited by Wnt/β-catenin signaling pathway, which this evidence suggesting application of Salinomycin as a therapeutic strategy for eradicating of OS-CSCs, while further clinical investigating are required [85, 185]. Based on available data, Salinomycin can reveal anticancer effects, which is implicated in autophagy. Authenticity of this supposes should be evaluated for achieving an appropriate anticancer [186]. Regarding the promising finding in preclinical investigation of Salinomycin, further clinical evaluation are recurrently required for clarifying its effectiveness and safety, where its toxicity issues such as amelioration of its systemic toxicity and optimization of dose should be taken into consideration [50, 184, 187].

#### Melatonin

Melatonin was greatly participated to reduce the invasion and migration of OS cells, which accordingly prevented onset and metastatic properties of OS in mice model. In addition, melatonin contributes to the inhibition of the sphere-forming in OS-CSCs, where have played regulatory roles for EMT marker in OS cells. Melatonin plays its key role in cancer stem cell inhibition by facilitating suppression of SOX9-mediated signaling in OS-CSCs [188].

#### Wogonin

Wogonin is known as compound derived from *Scutellaria baicalensis*, which have shown its anti-cancer effect (i.e., inhibition of angiogenesis, induction of apoptosis, inhibition of cancer growth etc.,) by modulating different signaling pathways such as PKB and AMPK pathways, and prevention of telomerase function, as well as p53-dependent/independent apoptosis [189, 190].

It has been revealed that Wogonin play its role as suppressor of stem cell-like traits with CD133 expression in OS cell and inhibits OS cell mobility in vitro through downregulation of matrix metallopeptidase-9 (MMP-9). Wogonin has been shown to inhibit sphere forming and reduce the size of spheres, leading to lower renewal capacity in CSC. Wogonin has been suggested to effective bioactive compound in preventing OS-CSCs in the bloodstream CSC OS metastases. Appling wogonin as a treatment approach or as a combination with other agents can be a good therapeutic approach, although more research is required in this regard [190].

#### Curcumin

Curcumin has been shown to play an anti-cancer effect in OS cells, its mediation can potentially be occurred via Notch-1 signaling inactivation, suggesting that curcumin is involved in upregulation of Notch-1 may be considered as a potential therapeutic strategy for OS [191]. It has been reported that apoptosis can be induced by Curcumin analog DK1 in human OS cells via mitochondria-dependent signaling, indicating its potential for future cancer treatment [192]. Moreover, Curcumin-loaded nanoparticles have been revealed to be involved in increasing apoptosis in OS cells [193].

## Conclusions

The regulation of intracellular markers including (IALDH), cell surface markers (CD133, CD117, CD44, CD 271, and Sca-1), stemness genes (Nestin, Sox2, OCT3/4, and Nanog), phenotypic evaluation (sphere forming assay), and side population (SP) cells are suggested to be useful in isolating OSCs of tumors. Among these proposed markers, the CD133, CD117, Stro-1 and ALDH with partial success in isolating CSCs has been confirmed as the most suitable markers for OSCs. Many therapeutic strategies have been highlighted to regulate the CSC-related pathways including Wnt, Hedgehog, Notch, PI3K/AKT, and TGFβ.

Targeting the Hh pathway is being explored to eradicate CSCs, especially SMO inhibitors for OS. Salinomycin and JW74 exhibited a pivotal role in inhibiting Wnt/β-catenin signaling as inhibitor of OSCs. However, gastrointestinal toxicity is a concern in terms of these inhibitors.

Epigenetic modulating intervention has attracted the most attention for targeted therapy of cancers. Present evidence supporting a role for imprinted gene TSSC3 pathway where reduced OSC phenotype, suggesting that targeting TSSC3 can be a new strategy for improving prognosis of OSC.

Targeting SDF-1, and of neutralizing CXCR4 represented a therapeutic strategy for cancer, both of which depicted a elevated expression level in many kinds of tumor cells.

A number of inhibitor has been entered in clinical trials as new therapeutic approaches for OS such as IGF1-R inhibitors, mTOR inhibitors, Multitarget inhibitors, and WNT/β-catenin inhibitor. Furthermore, a number of novel therapeutic agents used for OS by targeting osteoclasts and immune system based on the clinical trials. These drugs will provide a way to combine these targeting properties of the cells and their environment.

Multitarget drugs have revealed promise for OS as anti-angiogenesis therapy in clinical trial (i.e., Gefitinib). Anti-GD2, GD2Bi-aATC, and Mifamurtide, as well as stem cells and natural killer cells are other suggested therapy in clinical trials that potentially hamper cancer cells growth through harnessing of immune responses against tumors. It is difficult to treat a single molecule that reverses tumor characteristics. Thus, a combination of OSCs-targeting agents with chemoradiotherapy, surgery, or immunotherapy, and/or targeting the tumor microenvironment of CSCs, can be potentially effective in inhibiting tumor growth [50].
